# Supplementary material for: A minimal chemo-mechanical Markov model for rotary catalysis of F1-ATPase
Source: Nat Commun. 2026 Jun 15;17:5310. doi: 10.1038/s41467-026-73844-0 (PMC13272679; doi:10.1038/s41467-026-73844-0)
Supplement: Supplementary file 2 — Reporting Summary [file 41467_2026_73844_MOESM2_ESM.pdf]

nature portfolio

Corresponding author(s): Helmut Grubmüller

Last updated by author(s): Apr 23, 2026

Reporting Summary

Nature Portfolio wishes to improve the reproducibility of the work that we publish. This form provides structure for consistency and transparency in reporting. For further information on Nature Portfolio policies, see our Editorial Policies and the Editorial Policy Checklist.

Statistics

For all statistical analyses, confirm that the following items are present in the figure legend, table legend, main text, or Methods section.

n/a	Confirmed	
☐	☒	The exact sample size (*n*) for each experimental group/condition, given as a discrete number and unit of measurement
☒	☐	A statement on whether measurements were taken from distinct samples or whether the same sample was measured repeatedly
☒	☐	The statistical test(s) used AND whether they are one- or two-sided<br>*Only common tests should be described solely by name; describe more complex techniques in the Methods section.*
☒	☐	A description of all covariates tested
☒	☐	A description of any assumptions or corrections, such as tests of normality and adjustment for multiple comparisons
☐	☒	A full description of the statistical parameters including central tendency (e.g. means) or other basic estimates (e.g. regression coefficient) AND variation (e.g. standard deviation) or associated estimates of uncertainty (e.g. confidence intervals)
☒	☐	For null hypothesis testing, the test statistic (e.g. *F*, *t*, *r*) with confidence intervals, effect sizes, degrees of freedom and *P* value noted<br>*Give P values as exact values whenever suitable.*
☐	☒	For Bayesian analysis, information on the choice of priors and Markov chain Monte Carlo settings
☒	☐	For hierarchical and complex designs, identification of the appropriate level for tests and full reporting of outcomes
☒	☐	Estimates of effect sizes (e.g. Cohen's *d*, Pearson's *r*), indicating how they were calculated

Our web collection on statistics for biologists contains articles on many of the points above.

Software and code

Policy information about availability of computer code

Data collection	All data were collected using custom code (Python 3.9 and C).<br>The custom code developed in this study is publicly available on the Github repository YixinChen95/MarkovianF1 (https://github.com/YixinChen95/MarkovianF1), and archived at Zenodo (https://doi.org/10.5281/zenodo.19133448). The repository contains the C and Python scripts organized into four modules for Bayesian training of the Markov model, evaluation of model predictions, kinetic Monte-Carlo simulations, and hidden Markov analysis of the simulated trajectories, respectively. A README file provides instructions for installation and execution, and includes example workflows demonstrating the main functions of the code.
Data analysis	All data were collected using custom code (Python 3.9 and C).<br>The custom code developed in this study is publicly available on the Github repository YixinChen95/MarkovianF1 (https://github.com/YixinChen95/MarkovianF1), and archived at Zenodo (https://doi.org/10.5281/zenodo.19133448). The repository contains the C and Python scripts organized into four modules for Bayesian training of the Markov model, evaluation of model predictions, kinetic Monte-Carlo simulations, and hidden Markov analysis of the simulated trajectories, respectively. A README file provides instructions for installation and execution, and includes example workflows demonstrating the main functions of the code.

For manuscripts utilizing custom algorithms or software that are central to the research but not yet described in published literature, software must be made available to editors and reviewers. We strongly encourage code deposition in a community repository (e.g. GitHub). See the Nature Portfolio guidelines for submitting code & software for further information.

Data

Policy information about availability of data

All manuscripts must include a data availability statement. This statement should provide the following information, where applicable:

- Accession codes, unique identifiers, or web links for publicly available datasets
- A description of any restrictions on data availability
- For clinical datasets or third party data, please ensure that the statement adheres to our policy

Source Data are provided with this paper. The previously published atomic coordinates of F1-ATPase referred to in this study are available in the Protein Data Bank (PDB) under accession code 1BMF [https://doi.org/10.2210/pdb1BMF/pdb].

Research involving human participants, their data, or biological material

Policy information about studies with human participants or human data. See also policy information about sex, gender (identity/presentation), and sexual orientation and race, ethnicity and racism.

Reporting on sex and gender	Not applicable
Reporting on race, ethnicity, or other socially relevant groupings	Not applicable
Population characteristics	Not applicable
Recruitment	Not applicable
Ethics oversight	Not applicable

Note that full information on the approval of the study protocol must also be provided in the manuscript.

Field-specific reporting

Please select the one below that is the best fit for your research. If you are not sure, read the appropriate sections before making your selection.

☒ Life sciences ☐ Behavioural & social sciences ☐ Ecological, evolutionary & environmental sciences

For a reference copy of the document with all sections, see nature.com/documents/nr-reporting-summary-flat.pdf

Life sciences study design

All studies must disclose on these points even when the disclosure is negative.

Sample size	Sample size calculation is not applicable. The study relies on theoretical modeling and parameter inference using target values extracted from previously published literature, rather than statistical sampling of biological cohorts.
Data exclusions	No data were excluded from the analyses. This is a purely theoretical and computational modeling study based on previously published experimental data, not an experimental study involving raw data collection.
Replication	Replication is not applicable because this is a computational and theoretical study using Markov models and Bayesian parameter inference. It does not involve biological or technical replicates of experimental specimens.
Randomization	Randomized group allocation is not relevant to this study, as no experimental interventions or biological samples were involved. All analyses are based on theoretical physical models.
Blinding	Blinding is not relevant to this study because no experimental groups, animal models, or human subjects were used. The study relies entirely on computational modeling and mathematical simulations.

Reporting for specific materials, systems and methods

We require information from authors about some types of materials, experimental systems and methods used in many studies. Here, indicate whether each material, system or method listed is relevant to your study. If you are not sure if a list item applies to your research, read the appropriate section before selecting a response.

Materials & experimental systems

n/a	Involved in the study
☒	☐ Antibodies
☒	☐ Eukaryotic cell lines
☒	☐ Palaeontology and archaeology
☒	☐ Animals and other organisms
☒	☐ Clinical data
☒	☐ Dual use research of concern
☒	☐ Plants

Methods

n/a	Involved in the study
☒	☐ ChIP-seq
☒	☐ Flow cytometry
☒	☐ MRI-based neuroimaging

Plants

Seed stocks	Not applicable
Novel plant genotypes	Not applicable
Authentication	Not applicable
