# Supplementary material for: A minimal chemo-mechanical Markov model for rotary catalysis of F1-ATPase
Source: Nat Commun. 2026 Jun 15;17:5310. doi: 10.1038/s41467-026-73844-0 (PMC13272679; doi:10.1038/s41467-026-73844-0)
Supplement: Supplementary file 3 — Transparent Peer Review file [file 41467_2026_73844_MOESM3_ESM.pdf]

nature portfolio

Peer Review File

A Minimal Chemo-mechanical Markov Model for Rotary Catalysis of F_1-ATPase

Corresponding Author: Dr Helmut Grubmüller

This file contains all reviewer reports in order by version, followed by all author rebuttals in order by version.

Version 0:

Reviewer comments:

Reviewer #1

(Remarks to the Author)
Chen and Grubmuller provide here an in-depth analysis and F1-ATPase function and provide a chemomechanical Markov model that reproduces experimental data. Overall, I found the paper to be well-structured and it offers an interesting mechanistic insight into F1-ATPase catalysis to bring decades of structure/function studies together. I am not an expert in Markov modelling, I therefore cannot comment on the validity of the methods used. However, I do have some general comments regarding certain assumptions made in the text, as well as a few suggestions the authors may wish to consider prior to publication.

The authors draw upon a wide range of studies spanning decades and multiple organisms to present a comprehensive view of the F1-ATPase rotary catalytic mechanism. Although this analysis was extensive, I believe a few important nuances may have been overlooked:

(i) Sub stepping is not universal.
The authors reference work from the Noji, Yoshida, and Kinosita labs regarding 80/60° sub-stepping, which appears to be true for Bacillus strain PS3 and E. coli F1-ATPase. However, this behaviour is not universal. For example: the bovine and human enzymes exhibit a more complex sub stepping pattern (see Figure 1 https://doi.org/10.1073/pnas.1909407117) and the
Paracoccus denitrificans enzyme has yet to show any sub stepping (https://doi.org/10.1073/pnas.2003163117). Given these differences, I suggest the authors focus their modelling on a single species. Bacillus strain PS3 would be the likely target, as most of the data and ideas appear to be derived from studies on this species. My fear, is that incorporating data from multiple species may unnecessarily complicate their model.

(ii) ADP-, Pi-, and ADP+Pi-bound states.
In Section 2.1 (second paragraph), the model does not differentiate between ADP-, Pi-, and ADP+Pi-bound states. Our structural studies on Bacillus strain PS3 F1 (https://doi.org/10.1038/s41467-021-25029-0) identified two subtly distinct conformations we termed “half-open” and “half-closed”, with one hypothesis being they would preferentially bind ADP and ATP, respectively. This would nicely link to a binding change rotation model, where preferential binding on either side of the catalytic gate would likely be required for unidirectional rotation. Furthermore, the “half-open” 240° ADP+Pi-bound state seems unlikely to release ADP due to its partially closed nature and therefore, the statement that ADP/Pi release is a limiting step (also in Section 2.1) may lack clarity. My understanding is that, due to experimental limitations of the studies cited here, the precise timing of ADP release could not be determined using Cy3-ATP, and is instead inferred to occur between 240° and 320°, and not at a single dwell state.

(iii) Role of the gamma subunit.
In Section 2.1 (eighth paragraph), the authors explore the idea that the gamma subunit pushes the beta subunit during rotation. While this might be true, studies have also shown that large portions (and even the entire gamma subunit) can be removed (https://doi.org/10.1126/science.1151343 and https://doi.org/10.1126/science.1205510) and still rotate with ATP hydrolysis (albeit with significantly reduced rates). Furthermore, structural studies on these complexes (https://doi.org/10.1016/j.bbabio.2024.149521 and https://doi.org/10.1016/j.str.2023.12.014) suggest that the gamma subunit is not essential for opening the beta subunit. Would it be interesting to test whether the author's model remains valid in the

absence of gamma-beta subunit interactions?

(iv) Interpretation of structural states.
In Section 2.4 (final sentence), the authors state: "…because its predicted stable structures of the catalytic and ATP-waiting dwells agree with the classic "transition state" [5] and "ground- state" [4] structures of bovine F1-ATPase". I'm not sure I agree with this statement given that the authors are mainly interpreting Bacillus strain PS3 rotation data. The crystal structure in [5] is of a transition state but not of the PS3 F1-ATPase ATP binding dwell. Reinterpreting this crystal structure with single molecule rotation data on the bovine enzyme (Fig C https://doi.org/10.1073/pnas.1909407117), it may represent the transition state ~20 degrees after ATP binding?

(v) ATP-Waiting structure with one closed and two open beta subunits.
In the paragraph following Table 4, the authors describe a potential ATP-waiting structure with one closed and two open beta subunits. Structures of rotorless (when incubated with equimolar MgATP: https://doi.org/10.1016/j.str.2023.12.014) and axle-less (after incubation with MgAMP-PNP: https://doi.org/10.1016/j.bbabio.2024.149521) have both shown an open/half-open/closed structure, but again I doubt this half-open/closed conformation would allow for nucleotide exchange. Still the authors may wish to cite some of these papers, to at least confirm the possibility that this can occur and has been observed.

Alastair Stewart

(Remarks on code availability)

Reviewer #2

(Remarks to the Author)
Dear Authors,
This is an ambitious trial to elucidate and to explain the secret of the rotary motor mechanism. The simulation done was extensive and the results provided many constructive, useful, and suggestive information, especially for further researches on F-ATPase mechanism.
Problems:
The introduction contains only information from F-ATPase. V-ATPase has similar structure and similar mechanism. Further its chemo-mechanical coupling mechanism has been characterized very well and should be useful to consider this project. Its data and information should be cited, can be used, and must restrict the minimal conditions including the conformations in order to construct more plausible model of the chemo-mechanical coupling of rotary motors.
Details:
1. Transition rates were considered to be equal in both directions according to $k \propto \exp(-\Delta G‡/kBT)$, although the sources of such ΔG‡ values did not seem to be explained. It seems that they were obtained by the Baysian training fitting to the experimental data. Nucleotide affinities seemed also obtained as such. Then it is better to show such data and to discuss their probabilities. Nucleotide affinities can be compared with the experimental data, and the authors may have used such data for training; authors discussed some of the nucleotide affinities to specific β-conformations in supplementary materials. But the transition free energies were not available so far in experiments. So showing such data predicted in the simulations and discussing them with the available data from so far obtained in different experimental systems would be valuable and informative.
2. Authors defined free energies of the Markov states with several binding and conformational/interaction energies of γ and β. Authors should include the interaction energies of β and α, because they should also consist of main energies of the whole F-ATPase.
3. In page 8, authors described "A plausible explanation is that under this condition, a β-subunit is unlikely to open spontaneously and release the product after ATP hydrolysis, unless forced by the γ-subunit". Seemingly this is unbelievable because even without γ, F-ATPase hydrolyzes ATP. Is this consistent with the experimental data? Instead, the γ is thought to stabilize the β closed state by its interaction and then to stimulate the catalytic cycle.
4. As a reviewer, I could not follow the details of results and discussion sections. Since the sections 2.2 and 2.3 excluded the models as the plausible ones, it is better to remove them from the text: Because by utilizing the experimental data from V-ATPase, it seems possible and easy just by explaining in the introduction section to exclude such model. In addition, it must much more simplify the model explanation, I believe. Or it can be criticized that the experimental state of F-ATPase has not yet arrived at being subject and at being analyzed by simulations.
5. Following the above 4, the explanation of the model could be simplified by considering the available experimental data of rotary motors.
6. Sections 4 and 5 were persuasive. Considering the above claims, the explanation of the conditions and simulations could be revised extensively to be understood generally, easily and comprehensively.

(Remarks on code availability)

Reviewer #3

(Remarks to the Author)
Reviewer Report for "A Minimal Chemo-mechanical Markov Model for Rotary Catalysis of F1-ATPase"

This manuscript presents a modeling framework for F1-ATPase, in which the authors systematically construct, fit, and

evaluate multiple structural and conformational model variants against experimental data. Their approach begins with a literature-informed selection of degrees of freedom (DOFs), followed by a brute-force exclusion of implausible models. The remaining “most reasonable” variants are fitted to experimental data, with further verification steps applied to assess thermodynamic and kinetic consistency. This allows the authors to rule out several previously proposed models and identify model variants that align with all current experimental observations. From these results, the authors are able to draw several physical conclusions into the mechanics and thermodynamics of F1-ATPase functionality.

Overall the authors make their modeling decisions based on the relevant literature, and clearly explain their conclusions. As such, we strongly recommend this manuscript for publication.

Prior to publication, it would be good for the authors to think more broadly about generalizability and applicability of their methodology to studying other systems. While they suggest that their methodology may be effective in analysis of other enzymatic mechanisms, there may be barriers to the effective application to other systems due the many subjective modeling choices required by the researcher, leaving it up to the researcher to verify that the analysis has been systematic and complete. It also seems the process conducted here is very time intensive. In this case, the authors may consider revising the manuscript based on the points below in order to make their method more comprehensive and applicable in other systems.

Major Points

Although the manuscript cites extensive literature on the modeling choices, I believe readers unfamiliar with the specifics of F1-ATPase may feel lost regarding the essential differences between the model variants. Figure 1 appears insufficient to convey all the variants tested. A clearer comparison would help justify the selection of the “most reasonable” model.

There is a mixing of qualitative and quantitative methods for assessing the “most reasonable” model. While the authors do write down Bayes’ rule for the posterior over parameters, the downstream analysis focuses only on finding the maximum a posteriori estimate within each model variant. This is incomplete, as Bayes’ rule can also be applied to compare different model variants through marginalization over the parameter space. A discussion of such an approach would provide a principled framework for model comparison, reducing subjectivity and improving generalizability.

The manuscript separates the data used for fitting from that used for model validation, but the rationale for this division is unclear. Why are they treated differently? More explanation is needed—particularly as the Bayesian maximum a posteriori framework does not inherently require a training/validation split, unlike other machine learning classification methods. Additionally, are there more efficient or statistically principled approaches to identify model variants that fit all available data without relying so heavily on subjective modeling choices?

Minor Points

Typo: In Section 2.3 at one spot the text says “221” combinations instead of 211, the actual number.
Typo: Fig 3c subplot labels are wrong. The fourth subplot shows the one variant that doesn’t match the tryptophan data but is incorrectly labelled as ohc1*c2-m.2 instead of ohc1*c2-m.1.
I have questions about the different parameter sets and the four clusters discussed in Fig 2e. Do all 7500 parameter sets obtain similar posterior values?

(Remarks on code availability)

Reviewer #4

(Remarks to the Author)
The manuscript by Chen and Grubmuller develops a chemo-mechanical Markov model for F1-ATPase. The Markov model considers two substeps of the gamma rotation, three to four different conformational states of the beta-subunit, and three different nucleotide-bound states, as shown in Table 1. The model incorporates physical parameters, such as the conformational energy of the beta-subunit and the binding energies of ATP and ADP. The model was trained with experimental data that include [ATP]-dependent turnover rates, the chemo-mechanical coupling efficiencies, and the ATP/ADP titration curves. The model framework is general, and the approach is novel. It is a minimal model, but with sufficient details of the physicochemical properties. However, I have some major issues that need to be addressed, as described below.

1) It is described that the Markov model satisfies the local detailed balance. In this case, there is no net flux for the stationary distribution. In the Method, however, it is described that the turnover and rotation rates are given by the total net flux with the stationary distribution. How is the net flux generated? Please clarify this point.
2) In Fig. 1c and throughout the text, they only consider beta-subunits for different conformational states, as in the original Boyer’s binding change model. That assumption is made before the detailed structure of the protein is known. However,

crystal structures and their structural comparison clarified that although beta_TP and beta_DP have a similar conformation, the catalytic interface of these two is different, with a contribution from the neighboring alpha-subunit, such as "arginine finger", crucial for sensing the substrate and catalyzing its hydrolysis [Abrahams et al. Nature (1994); Okazaki and Takada, Structure (2011); Hayashi et al. JACS (2012)]. This point should be considered when the authors discuss the catalytic activity of the two closed conformations or when they introduce two different states for the closed state.
3) The ohc1c2 variants predict that the stable structure of the catalytic dwell is hc1c2, as shown in Table 4. However, a number of cryo-EM structures and single-molecule experiments suggest that the stable structure of the catalytic dwell is oc1c2 as the ground state structure, and the half-open (or half-closed) conformation is taken in the ATP-waiting dwell [Sobti et al. NatCommun (2021); Nakano et al. NatCommun (2023); Sugawa et al. PNAS (2016)]. This possibility needs to be explored in their model. A possible modification of the model is that the half-open conformation is only taken during the product release, but not in the ATP binding process. The current setup shown in Fig. 1e assumes that the "h" state exists in both binding and release events, which might not be true based on the structural comparison of alpha-beta subunits. Please see Fig. 7 of Sugawa et al. (https://www.pnas.org/doi/10.1073/pnas.1524720113). This point may also be related to the ignoring of the Pi release event in the model, which is a major driving force of the 40 degree rotation [Okazaki and Hummer, PNAS (2013)].
4) In Conclusion, it was described "for a nucleotide-bound beta-subunit, the catalytically active, closed conformation is energetically more favorable than the open and partially conformations." Where can I find the optimized values for supporting this?
5) In equation (10), the minus sign on the right-hand side seems unnecessary.

(Remarks on code availability)

Version 1:

Reviewer comments:

Reviewer #1

(Remarks to the Author)
The authors have addressed all my minor comments.

(Remarks on code availability)
I am not qualified to review this code

Reviewer #2

(Remarks to the Author)
Dear Authors,
Thank you for your extensive explanations and the revision. But I am sorry that I was rather disappointed by your responses. As a natural scientist, I would pursue to understand what is going on in our world, specifically the principal principle working in such marvelous enzyme machines of living organisms. I appreciate your efforts to understand the mechanism of F-ATPase, but as I have commented, I am afraid that the research level of F-ATPase has not arrived to discuss its mechanism. As you explain, the precise mechanisms of individual F-, V-, and A-ATPases would be different. But the principle of such energy transduction machineries would be expected to be the same. Scientists try to elucidate such general principles working in our world. If you are interested in the individual differences of them, you do not have to submit to Nat. Comm., a general science journal and rather should choose more specific journals.
What is known in V-ATPase mechanism (Arai et al., BBRC 533, 1413-1418 (2020)):
1) A and B subunits bind in the presence of nucleotides, especially with ATP; the affinity is weaker with ADP.
2) A or B alone has no ATP hydrolysis activity; A has ATP binding affinity.
3) A1B1 has high affinity for DF-axis; ATP (AMPPNP) stabilizes the A1B1DF complex strongly.
4) DF stabilizes A1B1 complex even without ATP.
Then we can speculate that A with ATP binds B, which in turn binds DF. A1B1DF hydrolyzes ATP to ADP, releasing Pi first in case of V-ATPase; A with ADP weakens the binding of B, thus of DF, releasing DF. DF floats thermally to the next ATP bound A1B1.
Is such mechanism known for F-ATPases? If so, then your research is valuable to understand the precise individual primary processes in the mechanism. Otherwise, it seems that your trials cannot specify the principal mechanism of the rotary ATPases. Therefore, I suggested that by including V-ATPase knowledges as universal principles of such rotary ATPases in the introduction section, the logics would become simpler and more easily understandable; just by renaming A, B, and DF to beta-, alpha-, and gamma-.
I suggest to address such principal principle of the rotary motors proposed for a specific V-ATPase as a precedent model, whether correct or wrong, and to try to discuss around such mechanism. Since I understood that the results in your section 4 and 5 seemed consistent with the proposed model, I expected that your research should provide great progress in our scientific knowledge. But your revision did not go into such discussion and just discussed about the specific individual processes, which shall give rather confusions in the scientific knowledge of rotary motors instead of giving development of clearer understanding what is going on in the rotary motors.
In this sense, I strongly oppose against publication of this manuscript as it is. I hoped that your works provide better understanding, but I am afraid that your manuscript as it is brings about only confusions in the research field of rotary motors.

(Remarks on code availability)

Reviewer #3

(Remarks to the Author)
The authors have addressed all comments. Ready for publication.

(Remarks on code availability)

Reviewer #4

(Remarks to the Author)
The authors sufficiently addressed the points raised. I believe it is now publishable.

(Remarks on code availability)

Version 2:

Reviewer comments:

Reviewer #2

(Remarks to the Author)
Dear Authors,
Thank you very much for your inclusive revision. I am happy to know that you understood my proposal of research attitude toward science. I fully agree with your comment aiming to elucidate the working principles under biological rotary motors. Touching about the precedent V-ATPase affinity change mechanism in introduction section made the logic clearer, simpler, and easily understandable. I appreciate your revisions in introduction and discussion.
At first stage of reviewing, I expected to shorten or to eliminate the 3-conformation model, because this part was not so much relevant to the principle of the mechanism and made the manuscript long and complicated. You kept this part in your previous revision, but I opposed against publication from different but more basic reason. Now since you agreed to include the V-ATPase affinity change mechanism in the introduction section, would you please reconsider this point? Other reviewers have agreed with publication as it was, and this point is not essential nor fatal for the improvement of the manuscript, therefore, I do not oppose against publication as it is, but I suggest you to reconsider whether extensive explanation of 3-variants model may bring about some confusion in readers.
Otherwise, I appreciate your revision and your present work. This must give researchers more profound and further progress in understanding the universal principle of the rotary motor mechanism.

(Remarks on code availability)

Open Access This Peer Review File is licensed under a Creative Commons Attribution 4.0 International License, which permits use, sharing, adaptation, distribution and reproduction in any medium or format, as long as you give appropriate credit to the original author(s) and the source, provide a link to the Creative Commons license, and indicate if changes were made.
In cases where reviewers are anonymous, credit should be given to 'Anonymous Referee' and the source.
The images or other third party material in this Peer Review File are included in the article's Creative Commons license, unless indicated otherwise in a credit line to the material. If material is not included in the article's Creative Commons license and your intended use is not permitted by statutory regulation or exceeds the permitted use, you will need to obtain permission directly from the copyright holder.
To view a copy of this license, visit https://creativecommons.org/licenses/by/4.0/

Point-by-point Responses

(for manuscript "A Minimal Chemo-mechanical Markov Model for Rotary Catalysis of F_1-ATPase")

Below, the Reviewers' comments are quoted (text in blue), followed by our replies (text in black). In the revised manuscript, we have highlighted all corresponding changes.

Reviewer #1

Chen and Grubmuller provide here an in-depth analysis and F1-ATPase function and provide a chemomechanical Markov model that reproduces experimental data. Overall, I found the paper to be well-structured and it offers an interesting mechanistic insight into F1-ATPase catalysis to bring decades of structure/function studies together.

Response: We thank Dr. Alastair Stewart for this very positive assessment.

I am not an expert in Markov modelling, I therefore cannot comment on the validity of the methods used. However, I do have some general comments regarding certain assumptions made in the text, as well as a few suggestions the authors may wish to consider prior to publication.

The authors draw upon a wide range of studies spanning decades and multiple organisms to present a comprehensive view of the F1-ATPase rotary catalytic mechanism. Although this analysis was extensive, I believe a few important nuances may have been overlooked:

(i) Sub stepping is not universal.
The authors reference work from the Noji, Yoshida, and Kinosita labs regarding 80/60° sub-stepping, which appears to be true for Bacillus strain PS3 and E. coli F1-ATPase. However, this behaviour is not universal. For example: the bovine and human enzymes exhibit a more complex sub stepping pattern (see Figure 1 https://doi.org/10.1073/pnas.1909407117) and the Paracoccus denitrificans enzyme has yet to show any sub stepping (https://doi.org/10.1073/pnas.2003163117). Given these differences, I suggest the authors focus their modelling on a single species. Bacillus strain PS3 would be the likely target, as most of the data and ideas appear to be derived from studies on this species. My fear, is that incorporating data from multiple species may unnecessarily complicate their model.

Response: This is an important point, and we fully agree. Our model was indeed developed to specifically describe the mechanism of F_1-ATPases from *E. coli* and *Bacillus* PS3 (EF_1 and TF_1), which exhibits the well-characterized 80° and 40° sub-steps. To ensure mechanistic consistency, the experimental data used for fitting model parameters ("training") and testing our model ("cross-validation") were therefore taken exclusively from studies on EF_1 and TF_1. We note that including EF_1 data is essential, because it offers unique and rich nucleotide titration data (binding affinities). These data are critical for determining the constraining the key energetic

parameters of our model; excluding them would leave those parameters highly under-determined.

Specifically, in line with the Reviewer's comment, we did not use data of F_1-ATPase from other species (*e.g.*, *Paracoccus denitrificans*, bovine, or human) for training or cross-validation, as these enzymes indeed show different sub-stepping behaviors. Notably, we referenced the broader F_1 literature only regarding the fundamental mechanistic assumptions (*e.g.*, the binding change principle) of our model, as these principles have been applied to the entire family before.

To make this scope clearer to the reader and avoid possible misunderstandings, we have now explicitly and globally stated our focus on EF_1 and TF_1 in the revised manuscript. Specifically, we have added a sentence in the Introduction section to highlight this focus (Page 3, highlighted in yellow); we have added Table 9 (Page 32, highlighted in yellow) to summarize the experimental data used for training and cross-validation of our model, highlighting the source of the data (*E. coli* or *Bacillus* PS3); and we have removed the original statement that compares our model predictions with bovine F_1 structures, also in response to the Reviewer's Comment (iv), as further detailed below.

(ii) ADP-, Pi-, and ADP+Pi-bound states.

In Section 2.1 (second paragraph), the model does not differentiate between ADP-, Pi-, and ADP+Pi-bound states. Our structural studies on Bacillus strain PS3 F1 (https://doi.org/10.1038/s41467-021-25029-0) identified two subtly distinct conformations we termed "half-open" and "half-closed", with one hypothesis being they would preferentially bind ADP and ATP, respectively. This would nicely link to a binding change rotation model, where preferential binding on either side of the catalytic gate would likely be required for unidirectional rotation. Furthermore, the "half-open" 240° ADP+Pi-bound state seems unlikely to release ADP due to its partially closed nature and therefore, the statement that ADP/Pi release is a limiting step (also in Section 2.1) may lack clarity. My understanding is that, due to experimental limitations of the studies cited here, the precise timing of ADP release could not be determined using Cy3-ATP, and is instead inferred to occur between 240° and 320°, and not at a single dwell state.

Response: Indeed, our model merges the ADP-, Pi-, and ADP+Pi-bound states into a single product-bound state "D". As we understand the Reviewer's comment, the Reviewer is concerned that this simplification might miss possibly relevant details of the multi-step nature of product release, where distinct conformations like the ones they identified ("half-open" and "half-closed") play sequential roles in first preparing for, and then executing, the final release of products. (Should we have missed the Reviewer's point, we would be grateful for clarification and a chance to reply).

In the revised Results section (Pages 5–6, highlighted in yellow), we now describe a central feature of our model more clearly, which is the conceptual un-coupling (for each individual β-subunit), of the binding state from the conformational state. Using the notation of our manuscript (see Table 1), this means the state of a β-subunit β_k $(k = 1, 2, 3)$ is specified by

two degrees of freedom, its binding state $\mathcal{B}^{(k)}$ and its conformational state $\mathcal{C}^{(k)}$. Further, we defined elementary transitions that connect these states where only *one* DOF changes at a time, *i.e.*, a conformational change and a chemical dissociation event are accounted for by two separate elementary transitions.

Due to this design choice, the multi-step product release process mentioned by the Reviewer is largely implicitly described by our model. For example, a β-subunit in the "D" binding state can indeed exist in multiple conformational states, such as "open" (o), "half-closed" (h), or "closed" (c_1/c_2). Therefore, the physically intuitive process of "first opening the catalytic site, then releasing the product" is represented within our model as a sequence of elementary transitions. For instance, a conformational transition step, (D, h) → (D, o), is followed by a chemical dissociation step, (D, o) → (E, o).

We would also like to clarify the terminology used in our manuscript. The phrase "rate-limiting product release step" was used in our original manuscript as a shorthand to refer to the effective kinetics of the chemical dissociation of our merged product state "D", which itself represents the slower of the individual ADP and Pi dissociation events. However, within the full catalytic cycle of our model, the overall rate of product release is not determined by a single pre-defined step but emerges from the collective kinetics of the entire multi-transition pathway. The true rate-limiting step for the cycle will be the slowest elementary transition within this pathway, which could be a conformational change or the chemical dissociation itself. We have now also clarified this issue in the revised manuscript.

Overall, this design decision is to prevent overfitting while retaining the crucial chemo-mechanical coupling: On the one hand, our model should be detailed enough to capture the *essential* kinetics of a conformationally-gated release process. On the other hand, by merging the final chemical product states (ADP, Pi, or ADP+Pi) into "D", we avoid the need to parameterize intermediate chemical states for which sufficiently accurate kinetic data are unavailable.

In addition to the added explanations (Pages 5–6, highlighted in yellow), to avoid confusion, we now distinguish the usage of two terms in our manuscript, using "product dissociation" to refer to the elementary chemical dissociation step, *e.g.*, (D, o) → (E, o), while using "product release" to refer to the full process that potentially involve multiple steps of conformational gating and chemical dissociation. Accordingly, the phrase "rate-limiting product release step" is replaced by "rate-limiting product dissociation step".

In support of this design decision, we want to emphasize that our model predictions are fully consistent with the Cy3-ATP experiment mentioned by the Reviewer. Indeed, our results (*e.g.*, Figure 3b and Supplementary Figure S4) show that in our model, the rate-limiting product release is not confined to a single dwell state, but is predicted to occur with significant probabilities at multiple dwell states, including 240° and 320°. These results support the view that product release is a multi-step process distributed over a range of dwells, rather than a single event at a specific dwell.

(iii) Role of the gamma subunit.
In Section 2.1 (eighth paragraph), the authors explore the idea that the gamma subunit pushes the beta subunit during rotation. While this might be true, studies have also shown that large portions (and even the entire gamma subunit) can be removed (https://doi.org/10.1126/science.1151343 and https://doi.org/10.1126/science.1205510) and still rotate with ATP hydrolysis (albeit with significantly reduced rates). Furthermore, structural studies on these complexes (https://doi.org/10.1016/j.bbabio.2024.149521 and https://doi.org/10.1016/j.str.2023.12.014) suggest that the gamma subunit is not essential for opening the beta subunit. Would it be interesting to test whether the author's model remains valid in the absence of gamma-beta subunit interactions?

Response: The Reviewer points to experimental data of γ-truncated or γ-depleted constructs and asks what our model would predict for these constructs. We agree that such data, along with the structural studies cited, points to allosteric interactions within the $\alpha_3\beta_3$ ring itself, which are not part of our model. It goes without saying that, in the context of our minimal model, only the main interactions will be considered, and others, assumed not essential for the main functional cycle, need to be neglected. For the case at hand, the observation that γ-depleted and γ-truncated constructs show greatly reduced hydrolysis rates and coupling efficiency, supports our assumption that the direct γ-β interaction are the main functional driver of chemo-mechanical coupling, while the allosteric communication within the $\alpha_3\beta_3$ ring represents a weaker, secondary effect.

For a more comprehensive discussion of how our model treats the different types of inter-subunit interactions, and how we have now expended our discussion in the revised manuscript, we would like to refer the Reviewer to our detailed response to Reviewer #2's Comments 2&3. Briefly, we have added a few sentences in the revised Results section (Pages 4, 6–7, highlighted in green) to explain our modeling of the inter-subunit interactions, clarifying our model focus on the primary mechanism; we have also added a brief discussion about the consequence and possible future extension (Page 20, highlighted in green).

Regarding the Reviewer's specific question of whether our model would still show some hydrolysis activity in the absence of γ-β interactions:

The answer is that it would to a certain extent show very low spontaneous hydrolysis activity, but it would miss the main determinant for the γ-depleted construct, which, in this case, *are* the weaker secondary allosteric interactions within the $\alpha_3\beta_3$ ring that our model by construction does not include. Modeling this secondary mechanism would be a fascinating extension for future work. A potential approach would be to introduce explicit interaction energies representing the allostery within the $\alpha_3\beta_3$ ring into the state free energies defined in our model. Such an extension would allow the model to both qualitatively and quantitatively account for the residual, inefficient rotation observed in γ-depleted constructs. This extension, however, is beyond the scope of our current manuscript.

(iv) Interpretation of structural states.

In Section 2.4 (final sentence), the authors state: "…because its predicted stable structures of the catalytic and ATP-waiting dwells agree with the classic "transition state" [5] and "ground-state" [4] structures of bovine F1-ATPase". I'm not sure I agree with this statement given that the authors are mainly interpreting Bacillus strain PS3 rotation data. The crystal structure in [5] is of a transition state but not of the PS3 F1-ATPase ATP binding dwell. Reinterpreting this crystal structure with single molecule rotation data on the bovine enzyme (Fig C https://doi.org/10.1073/pnas.1909407117), it may represent the transition state ~20 degrees after ATP binding?

Response: We thank the Reviewer for this comment. Also addressing the Reviewer's Comment (i), we have removed this statement, and are now refraining from using these bovine F_1 structures as extra support for any candidate model.

This comment also touches upon a deeper question about how our model predictions compare with the experimentally resolved crystal/cryoEM structures of F_1-ATPase. A more in-depth answer to this question, as well as the corresponding revisions of our manuscript, are detailed in our response to the related Comment 3 by Reviewer #4.

(v) ATP-Waiting structure with one closed and two open beta subunits.

In the paragraph following Table 4, the authors describe a potential ATP-waiting structure with one closed and two open beta subunits. Structures of rotorless (when incubated with equimolar MgATP: https://doi.org/10.1016/j.str.2023.12.014) and axle-less (after incubation with MgAMP-PNP: https://doi.org/10.1016/j.bbabio.2024.149521) have both shown an open/half-open/closed structure, but again I doubt this half-open/closed conformation would allow for nucleotide exchange. Still the authors may wish to cite some of these papers, to at least confirm the possibility that this can occur and has been observed.

Response: We thank the Reviewer for this constructive suggestion. We fully agree that these new references are highly relevant, providing important experimental support for the plausibility of the structures our model predicted. In the revised Discussion section, we now cite the suggested literature (Page 19, Refs. 69&70), in the context of discussing how recent cryoEM structures align with our model predictions (Pages 18–20, highlighted in purple). Indeed, these new references nicely adds to this new discussion (see also our response to Reviewer #4's Comment 3).

Also related to our response to Reviewer #4's Comment 3 below, and in response to this Reviewer's specific comment about whether the half-open/half-closed structure observed in experiments would allow for nucleotide exchange, we emphasize that the conformations of individual β-subunits (o, h, c/c_1/c_2) are defined in our model as *functional* states rather than as *structural* states and, therefore, they do not directly correspond to the experimentally resolved atomistic "snapshots" (Page 4, highlighted in purple). Particularly, the h state defined in our model represents the kinetically relevant intermediate of a β-subunit that facilitates product dissociation; it does not necessarily correspond to the experimentally observed half-open/closed structure. Given that product dissociation is observed to be rate-limiting, this functional h state

must, by thermodynamic principles, represent a meta-stable conformation that controls the free energy barrier for product dissociation.

Therefore, we think that our model does not contradict the intuition that the observed half-open/half-closed structure might be too "closed" for rapid nucleotide exchange. In fact, the actual conformation that controls product dissociation in reality (labelled *h* in our model) might look different from the observed half-open/half-closed structure. For example, the actual conformation of the *h* state might be somewhere between the observed half-open/half-closed and fully open structures.

Reviewer #2

This is an ambitious trial to elucidate and to explain the secret of the rotary motor mechanism. The simulation done was extensive and the results provided many constructive, useful, and suggestive information, especially for further researches on F-ATPase mechanism.

Response: We thank the Reviewer for this very positive and encouraging feedback.

The introduction contains only information from F-ATPase. V-ATPase has similar structure and similar mechanism. Further its chemo-mechanical coupling mechanism has been characterized very well and should be useful to consider this project. Its data and information should be cited, can be used, and must restrict the minimal conditions including the conformations in order to construct more plausible model of the chemo-mechanical coupling of rotary motors.

Response: We thank the Reviewer for suggesting to consider information on V-ATPase more extensively in our manuscript. Notably, this suggestion is in contrast to the comments of Reviewer #1 (see particularly Comment (i)), who actually was very skeptical about mixing data from different species even within the F-ATPase family, and therefore suggested to focus on F-ATPase from one bacterial species only, because mechanistic details of other F-ATPases might already be different. Hence, we were a bit torn back and forth between these two directions.

To decide how to proceed, we have now conducted an in-depth literature review on the relationship between F- and V-type ATPases. Obviously, the two ATPase families share a common evolutionary ancestor, a similar structural architecture, and the core principle of rotary catalysis. However, the differences between the two ATPase families are much more pronounced than those amongst the different F-ATPase species (Iino *et al.*, *Iubmb Life*, 2014; Muench *et al.*, *Q. Rev. Biophys.*, 2011; Stewart *et al.*, *Curr. Opin. Struct. Biol.*, 2014). In particular, V-ATPases are seen to rotate in 120° steps (Minagawa *et al.*, *J. Biol. Chem.*, 2013) without the 80° and 40° sub-steps that are an essential part of our model. Combined with other differences in, *e.g.*, primary physiological function (ATP synthesis vs. dedicated proton

pumping), subunit complexity, and regulatory mechanisms, we think that the thermodynamic energy landscapes and kinetic parameters for the two motors are too different for an attempt to describe them with one unified model, even at the rather coarse-grained level of our approach.

For these reasons, we would prefer to restrict our model to F-ATPases in the present study. Nevertheless, we are intrigued by the challenge of adapting our framework to the broader rotary ATPase families – including V- and A-type ATPases – in the future, which could reveal unifying principles of energy conversion across these similar molecular machines. We have added a brief discussion in the revised manuscript that places our work in a broader context of rotary ATPase families, in particular V-ATPases (Page 21, highlighted in yellow).

1. Transition rates were considered to be equal in both directions according to k ∝ exp(-ΔG‡/kBT), although the sources of such ΔG‡ values did not seem to be explained. It seems that they were obtained by the Baysian training fitting to the experimental data. Nucleotide affinities seemed also obtained as such. Then it is better to show such data and to discuss their probabilities. Nucleotide affinities can be compared with the experimental data, and the authors may have used such data for training; authors discussed some of the nucleotide affinities to specific β-conformations in supplementary materials. But the transition free energies were not available so far in experiments. So showing such data predicted in the simulations and discussing them with the available data from so far obtained in different experimental systems would be valuable and informative.

Response: We thank the Reviewer for this detailed comment about free energy barriers ($\Delta G^{\ddagger}$) and nucleotide affinities in our model. It highlights several points that we may have not explained sufficiently clearly in our original manuscript, mostly regarding the definition of our model parameters and the Bayesian training procedure. We have therefore now substantially revised the Results and Methods sections to provide a more structured, detailed and comprehensive explanation, as summarized below.

As the Reviewer correctly points out, the heights ($\Delta G^{\ddagger}$) of the free energy barriers are defined as adjustable parameters of our model. Additionally, we also defined several free energy contributions as free parameters, such as conformational free energies and *microscopic* binding free energies assigned to each conformational state of individual β-subunits; for each Markov state, these add up to the total free energy of that state. To clearly summarize these parameters, we refer to the new Table 7 in the revised manuscript (Page 27, highlighted in yellow).

It is also correct that our parameters were not assumed *a priori* but were determined by our Bayesian training procedure to best fit (globally) all the considered experimental data. Indeed, as noted by the Reviewer, the obtained free energies and free energy barriers govern the transition rates in our model. However, contrary to what the Reviewer seems to have concluded from our text, we do not assume all forward and backward rates to be equal, which would be a mistake. Rather, our model strictly enforces local detailed balance (microscopic reversibility). Under this condition, following standard transition state theory, the ratio of the forward and backward rate constants (k_{f}, k_{b}) for any given transition is determined by the free energy

difference ΔG between the two connected states, such that $k_f/k_b = \exp(-\Delta G/k_B T)$, while their absolute values (generally differing from each other) are mainly given by the free energy barrier height $\Delta G^{\ddagger}$ relative to the free energy G of the respective state. The principles and exact definitions governing these rates are now more concisely summarized in the new Table 5 in the revised manuscript (Page 25, highlighted in yellow).

Regarding the nucleotide affinities mentioned by the Reviewer (*apparent* binding affinities), we note the important distinction between the *apparent* binding affinities and the *microscopic* binding free energies that we have defined as model parameters, which may have not become sufficiently clear in our original manuscript. Indeed, our model is trained to reproduce the experimental titration curves; to this end, the *apparent* binding affinities are obtained by fitting the model-predicted titration curves to a simple equilibrium binding model (see Methods Section 4.6, particularly Equation (30), Page 35). Thus, these *apparent* binding affinities are emergent properties calculated from our model rather than fundamental adjustable parameters. We had included a detailed derivation for the relation between the *apparent* binding affinities and the *microscopic* binding free energies in the original submission, but only in the Supplementary Materials – easily overlooked, yet still spotted by the Reviewer. To better emphasize this important distinction, we have now added further clarifications to the revised Results and Methods sections (Pages 8, 12, 34–36, highlighted in yellow).

In addition to these specific revisions, we have globally revised the entire Results Sections 2.1–2.3 (Pages 4–13, underlined) to better guide the readers through these logics; and we have substantially revised and extended the Methods Sections 4.1–4.3 and 4.6 (Pages 22–29, 34–36) to provide all detailed information in a more clear and structured manner.

Finally, we fully agree with the Reviewer that it would be very useful to compare the values of the trained parameters with experimental data. However, as the Reviewer also correctly points out, measurements of microscopic transition rates that would directly correspond to the free energy barriers in our model would be quite challenging, and we are unaware of any published suitable data. Nevertheless, we have now included the relevant distributions of the trained parameter values within the Supplementary Materials (Supplementary Figures S11–S14) to enable comparison with potential future measurements.

2. Authors defined free energies of the Markov states with several binding and conformational/interaction energies of γ and β. Authors should include the interaction energies of β and α, because they should also consist of main energies of the whole F-ATPase.

3. In page 8, authors described "A plausible explanation is that under this condition, a β-subunit is unlikely to open spontaneously and release the product after ATP hydrolysis, unless forced by the γ-subunit". Seemingly this is unbelievable because even without γ, F-ATPase hydrolyzes ATP. Is this consistent with the experimental data? Instead, the γ is thought to stabilize the β closed state by its interaction and then to stimulate the catalytic cycle.

Response: We thank the Reviewer for these two insightful comments, which also go in line

with Reviewer #1's Comment (iii) and Reviewer #4's Comment 2. These comments address the important question of which are the main inter-subunit interactions that govern the tight chemo-mechanical coupling of F_1-ATPase, and therefore need to be included within our minimal model, and which interactions can be neglected in the attempt to single out the primary mechanism. Hence, our response below refers to the comments of the three Reviewers.

To design a minimal yet physically realistic model, we first considered the different inter-subunit interactions and their relative importance for achieving tight chemo-mechanical coupling. To answer the Reviewers' comments, it helps to distinguish between three key types of interactions:

(1) Direct, camshaft-like forces exerted by the central γ-rotor on the β-subunits (γ-β interactions);
(2) The interactions between the α- and β-subunits mentioned by the Reviewer. These can be grouped into:
    a. Direct, local interactions between adjacent α- and β-subunits (local α-β interactions). Such direct contacts at the catalytic interface, *e.g.*, involving the "arginine finger", define the local environment of each β-subunit.
    b. Long-range allosteric effects within the $\alpha_3\beta_3$ ring, *i.e.*, between different catalytic sites across the entire $\alpha_3\beta_3$ hexamer.

In Comment 2, the Reviewer suggests we should include α-β interactions. We fully agree, and we note the foundational role of local α-β interactions, which define the energy landscape of the catalytic sites. In our model, these interactions are accounted for implicitly within the very definition of the β-subunit's conformational states. More specifically, the energy contributions of the local α-β interactions are part of the conformational free energies and binding free energies of the respective β-subunits. These parameters should therefore be understood as the effective energies of a β-subunit in a certain conformation within its local environment, mainly defined by the interactions with the adjacent α-subunits.

The long-range allostery within the $\alpha_3\beta_3$ ring, in contrast, are indeed not included within our minimal model, as we consider them less important for an understanding and proper description of the main mechanism. Our assumption is supported by the observation that γ-depleted and γ-truncated F_1-ATPase, as also pointed out by Reviewer #1's Comment (iii), shows much lower residual hydrolysis activity than the intact F_1-ATPase, in line with our statement that a β-subunit is much less likely to open spontaneously "unless forced by the γ-subunit". This observation, indeed, strongly suggests that the γ-β interactions are the primary driver of tight and efficient coupling, whereas the long-range allostery within the $\alpha_3\beta_3$ ring constitute a much weaker, secondary mechanism, which only becomes important for the low residual activity of γ-depleted and γ-truncated F_1-ATPase constructs.

To make this modeling philosophy and its justification clearer, we have added a few sentences in the revised Results section (Pages 4, 6–7, highlighted in green) to explain our modeling of the inter-subunit interactions, clarifying our model focus on the primary mechanism; we have

also added a brief discussion about the consequence and possible future extension (Page 20, highlighted in green).

4. As a reviewer, I could not follow the details of results and discussion sections. Since the sections 2.2 and 2.3 excluded the models as the plausible ones, it is better to remove them from the text: Because by utilizing the experimental data from V-ATPase, it seems possible and easy just by explaining in the introduction section to exclude such model. In addition, it must much more simplify the model explanation, I believe. Or it can be criticized that the experimental state of F-ATPase has not yet arrived at being subject and at being analyzed by simulations.

5. Following the above 4, the explanation of the model could be simplified by considering the available experimental data of rotary motors.

6. Sections 4 and 5 were persuasive. Considering the above claims, the explanation of the conditions and simulations could be revised extensively to be understood generally, easily and comprehensively.

Response: We thank the Reviewer for the feedback on our manuscript's readability. Also in response to the Reviewer's Comment 1, we have thoroughly revised the manuscript to strictly distinguish between the narrative flow and technical documentation. The entire Results Sections 2.1–2.3 (Pages 4–13, underlined) have been rewritten to focus on the essential logics and physical conclusions. Meanwhile, all supporting mathematical formulas, definitions, and computation details have been consolidated into the revised Methods Sections 4.1–4.7 (Pages 22–29, 34–38, underlined). Particularly, we have extensively utilized summary tables to organize this dense information systematically, facilitating easier lookup and comparison (Tables 5–13).

We have discussed the suggestion to use V-ATPase data to simplify the model selection process above. Given the substantial functional and mechanistic differences between F- and V-ATPases, we are concerned that directly applying constraints from one system to the other would compromise the data-driven nature of our study, possibly resulting in a "chimeric model" that fits neither of the two.

Regarding the suggestion to remove some of our tentative models from the text, we would prefer not to. We think that the systematic, step-by-step exclusion of the *ohc*-variants based on a rigorous comparison with F-ATPase-specific data is not just a less important step of our model construction; rather, we consider it a central and quite unexpected finding that a model with only three β-subunit conformations is fundamentally incompatible with the known properties of F_1-ATPase, which we would prefer not to hide. We have improved our revised manuscript to clarify this important point and the underlying logical flow.

Reviewer #3

Reviewer Report for "A Minimal Chemo-mechanical Markov Model for Rotary Catalysis of F1-ATPase"

This manuscript presents a modeling framework for F1-ATPase, in which the authors systematically construct, fit, and evaluate multiple structural and conformational model variants against experimental data. Their approach begins with a literature-informed selection of degrees of freedom (DOFs), followed by a brute-force exclusion of implausible models. The remaining "most reasonable" variants are fitted to experimental data, with further verification steps applied to assess thermodynamic and kinetic consistency. This allows the authors to rule out several previously proposed models and identify model variants that align with all current experimental observations. From these results, the authors are able to draw several physical conclusions into the mechanics and thermodynamics of F1-ATPase functionality.
Overall the authors make their modeling decisions based on the relevant literature, and clearly explain their conclusions. As such, we strongly recommend this manuscript for publication.

Response: We are very grateful to the Reviewer for the strong support.

Prior to publication, it would be good for the authors to think more broadly about generalizability and applicability of their methodology to studying other systems. While they suggest that their methodology may be effective in analysis of other enzymatic mechanisms, there may be barriers to the effective application to other systems due the many subjective modeling choices required by the researcher, leaving it up to the researcher to verify that the analysis has been systematic and complete. It also seems the process conducted here is very time intensive. In this case, the authors may consider revising the manuscript based on the points below in order to make their method more comprehensive and applicable in other systems.

Response: We thank the Reviewer for this comment on the generalizability of our methodology. The Reviewer raises two main points, namely, the potential subjectivity of our modeling choices, and the time-intensive nature of our analysis. Below, we will address these two separately.

Regarding the Reviewer's concern about subjective modeling choices, we realize that our original manuscript did not sufficiently highlight how our systematic approach is, in fact, designed to navigate these choices objectively and systematically. As also reflected in Comments 4 – 6 by Reviewer #2 and our reply above, we were prompted to considerably revise the description of our modeling choices in the Results Section 2.1 (Pages 4–7, underlined).

Of course, as the Reviewer will certainly agree, ***any*** model requires choices of what to include and what to neglect, which need to be justified. Obviously, this task becomes more challenging with increasing complexity of the object at hand.

As we understand it, the Reviewer perceives two potential levels of subjectivity. The first level

regards decisions about fundamental model assumptions, such as choices of key degrees of freedom (DOFs). These choices are necessarily system-specific and informed by previous studies available for a given system. Notably, as our description of the step-by-step refinement of these choices by our systematic model comparison strategy ("training + cross-validation") shows, such choices can and do turn out to be inadequate *a posteriori*, *i.e.*, as it turned out that considering only three main conformations of the β-subunit made it impossible to obtain agreement with all relevant experimental data. This and similar results provide examples for an objective, data-driven criterion for which DOFs need to be included as essential parts of the model, thereby minimizing what would otherwise be a subjective choice.

The second level of subjectivity, which the Reviewer addresses below ("Major points"), concerns our model comparison strategy. As we justify in our detailed response below, we believe our "training + cross-validation" strategy is a pragmatic, principled, and objective strategy for model comparison in a study at this level of complexity.
Regarding the Reviewer's concern about the time-intensive nature of our analysis process, we agree that our model construction and validation required quite a bit of effort indeed. We would like to note, however, that while computationally expensive, our extensive model parameter search was essential for our key finding regarding the minimal number of β-subunit conformations as mentioned above. Very much like the prototype of a new car is unaffordably expensive, while serial production yields amazing efficiencies, we are convinced that our "prototypic" study paves the way for future studies with substantially increased efficiency.

We also think it would be misleading to consider the theoretical framework described here as a "black-box" automated tool; rather, it should serve as an example for a general research strategy providing conceptual guidance for researchers tackling other complex enzymatic systems.

In response to the Reviewer's comments, we have added two paragraphs in the Discussion section to better articulate our methodological philosophy and its role in minimizing subjectivity (Pages 20–21, highlighted in blue).

Major points:

Although the manuscript cites extensive literature on the modeling choices, I believe readers unfamiliar with the specifics of F1-ATPase may feel lost regarding the essential differences between the model variants. Figure 1 appears insufficient to convey all the variants tested. A clearer comparison would help justify the selection of the "most reasonable" model.

Response: We thank the Reviewer for this helpful suggestion to improve the clarity of our presentation. Indeed, a more direct, visual comparison of the key model variants is certainly very helpful for the reader to follow the logic of our systematic model selection process. We have therefore revised the manuscript along these lines:

(1) We have added a new subplot (f) to Figure 1 (Page 41) that graphically illustrates the γ-β restrictions specifying a model variant. As these restrictions are the primary

hyperparameters that define and differentiate the variants, this new visual aid provides an intuitive overview of their core differences, directly addressing the Reviewer's feedback about the insufficiency of the original Figure 1.

(2) We have substantially revised the Results Section 2.1 (Pages 4–7, underlined) to better guide the reader through our model construction process. The revised text provides a more structured explanation about our model, and explicitly connects our narrative to both the new graphical illustration in Figure 1f and the detailed definitions of several model variants in Tables 2 and 3. This creates a clearer, step-by-step explanation of the essential differences between the variants before we proceed with their evaluation and selection.

(3) We have added Table 6 to summarize the hyperparameters (Page 26, highlighted in yellow).

These combined revisions will certainly make our model selection process more transparent and easier for the reader to follow.

There is a mixing of qualitative and quantitative methods for assessing the "most reasonable" model. While the authors do write down Bayes' rule for the posterior over parameters, the downstream analysis focuses only on finding the maximum a posteriori estimate within each model variant. This is incomplete, as Bayes' rule can also be applied to compare different model variants through marginalization over the parameter space. A discussion of such an approach would provide a principled framework for model comparison, reducing subjectivity and improving generalizability.

The manuscript separates the data used for fitting from that used for model validation, but the rationale for this division is unclear. Why are they treated differently? More explanation is needed—particularly as the Bayesian maximum a posteriori framework does not inherently require a training/validation split, unlike other machine learning classification methods. Additionally, are there more efficient or statistically principled approaches to identify model variants that fit all available data without relying so heavily on subjective modeling choices?

Response: We thank the Reviewer for these methodological comments addressing the overarching topic of model comparison and selection. These comments highlighted that the rationale for our strategy was not described sufficiently clearly in our original manuscript. In response, we have substantially revised the manuscript, as summarized below.

Addressing the Reviewer's specific points:

As we understand it, the core of these comments is the question whether our current model comparison strategy ("training + cross-validation") is appropriate, and if it should be replaced by a more formal Bayesian model comparison, such as evaluating the marginal likelihood.

First, we would like to clarify that our Bayesian training approach is more extensive than finding a single *MAP* estimate of the parameters. For each model variant, through thousands of

independent optimization runs from different initial points, we broadly sampled the high-posterior regions of the parameter space. This extensive sampling revealed a complex posterior landscape with multiple clusters of parameter sets yielding similarly high posterior probabilities (as shown in Fig. 2d, e), all of which were used for cross-validation. Therefore, our analysis of each model variant is not restricted to a single *MAP* estimate, but takes full account of an ensemble of parameter sets sufficiently representing the posterior landscape. We have now clarified this point in the revised Results and Methods sections (Pages 8, 30, 31, 33, 34; multiple relevant sentences highlighted in blue).

Regarding the Reviewer's suggestion of using a formal Bayesian method for model comparison, evaluating the marginal likelihood would indeed be the gold standard. However, for the case at hand we faced significant computational hurdles; specifically, for our model we were unable to achieve sufficient sampling of the posterior distribution to determine the marginal likelihood in absolute terms, as would be required. The main bottleneck here is the high computational cost of evaluating the likelihood function for each single parameter set, which requires solving the master equation for a system with up to several hundred states.

We therefore resorted to cross-validation, which is a pragmatic and widely used alternative for comparing complex models. This approach has the additional benefit of assessing the predictive power of the models. We agree with the Reviewer that a fully Bayesian approach should not require cross-validation – unless there are uncertainties in the modelling of the experiments by the likelihood function. These uncertainties would not be detected by comparing the marginal likelihoods; in contrast, cross-validation has a fair chance to detect such "unknown unknowns". Hence, we argue that cross-validation is not just a pragmatic choice due to computational limits, but offers the advantage of an additional check of the underlying physical assumptions.

Addressing the Reviewer's question of why the training data and validation data are treated differently, our division was a deliberate choice, based on the heterogeneous nature of the data, which describe different aspects of F_1-ATPase function:

The training data (turnover rates, chemo-mechanical coupling efficiency, and nucleotide titration curves) consist of quantitative measurements that characterize the macroscopic properties of the system. These data are well-suited for direct inclusion in a likelihood function for rigorous parameter fitting.

In contrast, the validation data (the population shift between dwells, and the orientations at which the major catalytic steps occur) are semi-quantitative or qualitative, revealing the system's mechanistic details on a more microscopic level. Formulating a precise, statistically rigorous likelihood term for these observations would be challenging and could introduce further subjective assumptions and complication. We therefore decided to use these data for cross-validation instead, which does not require such quantitative modeling.

Further, we found that our "training + cross-validation" strategy is also helpful in model diagnosis. If instead all data were used in fitting and it failed, it would be more difficult to

pinpoint the source of the problem. In contrast, with the experimental data separated into training and validation sets, if a model successfully fits the training data but fails to predict the validation data, we can conclude that its description of the underlying chemo-mechanical coupling mechanism is flawed, even if its overall steady-state behavior appears correct.

We have added a new Methods Section 4.4 ("Systematic model comparison strategy", Pages 30–31, underlined) to articulate these arguments for using cross-validation and splitting training/validation datasets.

Finally, the Reviewer asks about more efficient or statistically principled approaches for model selection. While we hope to have convinced the Reviewer that a textbook-like statistically principled approach would currently be unfeasible due to the complex nature of F_1-ATPase and experimental uncertainties, we offer some speculations regarding possible future directions to enhance efficiency. For general model comparison problems of complex systems, one might find ideas from the intersection of Bayesian statistics and machine learning. For instance, one might use simulation-based inference or machine learning to build computationally inexpensive "surrogate models" of the likelihood function. Such advances might make computing the marginal likelihood of a model variant more tractable for systems of this complexity, and this marginal likelihood could provide an additional quantitative criterion for automatic model comparison, thus complementing cross-validation.

Minor points: Typo: In Section 2.3 at one spot the text says "221" combinations instead of 211, the actual number.
Typo: Fig 3c subplot labels are wrong. The fourth subplot shows the one variant that doesn't match the tryptophan data but is incorrectly labelled as ohc1*c2-m.2 instead of ohc1*c2-m.1.
I have questions about the different parameter sets and the four clusters discussed in Fig 2e. Do all 7500 parameter sets obtain similar posterior values?

Response: We thank the Reviewer for catching these two typos. We have corrected them in the revised manuscript. Specifically, we have further corrected the number of combinations for *ohc*-variants to 665 (see the revised Methods section, Page 26), to ensure consistency of such estimations for both *ohc*- and ohc_1c_2-variants.

Regarding the Reviewer's final question, the quick answer is: Yes, they do. In more detail: All the parameter sets selected and shown in Fig. 2e achieved similarly high posterior probabilities, indicating that they are all statistically plausible solutions. This finding suggests that the posterior landscape is quite complex, likely containing multiple distinct high-probability regions (clusters of slightly different functional mechanisms) or, alternatively and less likely, a broad plateau. These different clusters, while fitting the training data similarly well, predict different mechanistic details of the catalytic pathways, which is why we have carried them forward for further evaluation in the cross-validation step. These above points have been clarified in the revised manuscript (Page 8, highlighted in blue).

Reviewer #4

The manuscript by Chen and Grubmuller develops a chemo-mechanical Markov model for F1-ATPase. The Markov model considers two substeps of the gamma rotation, three to four different conformational states of the beta-subunit, and three different nucleotide-bound states, as shown in Table 1. The model incorporates physical parameters, such as the conformational energy of the beta-subunit and the binding energies of ATP and ADP. The model was trained with experimental data that include [ATP]-dependent turnover rates, the chemo-mechanical coupling efficiencies, and the ATP/ADP titration curves. The model framework is general, and the approach is novel. It is a minimal model, but with sufficient details of the physicochemical properties. However, I have some major issues that need to be addressed, as described below.

Response: We thank the Reviewer for appreciating the novelty of our approach.

1) It is described that the Markov model satisfies the local detailed balance. In this case, there is no net flux for the stationary distribution. In the Method, however, it is described that the turnover and rotation rates are given by the total net flux with the stationary distribution. How is the net flux generated? Please clarify this point.

Response: We thank the Reviewer for this question, which allows us to clarify a fundamental concept of our modeling framework.

The Reviewer correctly states that our model enforces ***local*** detailed balance for every individual transition between two Markov states. In this sense, all transitions are reversible, and, for each transition, the forward and backward rate coefficients are related by the free energy difference between the two connected states, as also described in our reply to Reviewer #2 (Comment 1) above.

However, our model does not obey ***global*** detailed balance, which indeed would imply "no net flux for the stationary distribution". Rather, our model treats F_1-ATPase as an open system in contact with external ATP and ADP reservoirs, assuming that the concentrations of ATP and ADP in the solution change only slowly relative to the turnover rate of F_1-ATPase, which, given the physiological concentrations, is a very good approximation. As a result, during one catalytic cycle (a 120°-rotation of the γ subunit), the free energy of the system drops by about 13 kcal/mol, which is the free energy released by ATP hydrolysis. This fee energy is conceptually provided if, during each catalytic cycle, one ADP molecule of the chemical reservoir is replaced by one ATP molecule. Because the latter is not explicitly included within our model (nor does it have to), the free energy goes continuously "downhill" by 13 kcal/mol pre ATP hydrolyzed. Accordingly, the system is never in equilibrium (nor is an active F_1-ATPase system hydrolyzing or synthesizing ATP in reality), but one may say it is in a constant net flux condition.

To make this point clearer also for the readers, we have added a few clarifying sentences in the Results and Methods sections (Pages 6, 27–28; highlighted in blue).

2) In Fig. 1c and throughout the text, they only consider beta-subunits for different conformational states, as in the original Boyer's binding change model. That assumption is made before the detailed structure of the protein is known. However, crystal structures and their structural comparison clarified that although beta_TP and beta_DP have a similar conformation, the catalytic interface of these two is different, with a contribution from the neighboring alpha-subunit, such as "arginine finger", crucial for sensing the substrate and catalyzing its hydrolysis [Abrahams et al. Nature (1994); Okazaki and Takada, Structure (2011); Hayashi et al. JACS (2012)]. This point should be considered when the authors discuss the catalytic activity of the two closed conformations or when they introduce two different states for the closed state.

Response: We thank the Reviewer for raising this mechanistic point, and we fully agree that the effects of the differences at the α-β interfaces should be considered. In fact, these effects are not omitted in our model, but are implicitly accounted for by the different conformational states (and their free energies) of the β-subunits. For example, the different thermodynamic properties of the two closed states (conformational free energies and binding free energies) also capture the different functional consequences of these varying local α-β interactions at the catalytic site. This approach is in line with our ambition to construct a minimal model, which is not meant to reflect structural details. For a more in-depth explanation, we refer the Reviewer to our response to a similar question by Reviewer #2 (Comments 2 & 3).

To make this point clearer to the readers, we have added several sentences in the Results section that clarify these points (Pages 4, 6–7; highlighted in green).

3) The ohc1c2 variants predict that the stable structure of the catalytic dwell is hc1c2, as shown in Table 4. However, a number of cryo-EM structures and single-molecule experiments suggest that the stable structure of the catalytic dwell is oc1c2 as the ground state structure, and the half-open (or half-closed) conformation is taken in the ATP-waiting dwell [Sobti et al. NatCommun (2021); Nakano et al. NatCommun (2023); Sugawa et al. PNAS (2016)]. This possibility needs to be explored in their model. A possible modification of the model is that the half-open conformation is only taken during the product release, but not in the ATP binding process. The current setup shown in Fig. 1e assumes that the "h" state exists in both binding and release events, which might not be true based on the structural comparison of alpha-beta subunits. Please see Fig. 7 of Sugawa et al. (https://www.pnas.org/doi/10.1073/pnas.1524720113). This point may also be related to the ignoring of the Pi release event in the model, which is a major driving force of the 40 degree rotation [Okazaki and Hummer, PNAS (2013)].

Response: We thank the Reviewer for this comment, which points to a seeming discrepancy between the "stable structures" predicted by our model and recent cryoEM studies [Sobti et al. (2021); Nakano et al. (2023)]. It prompted us to think deeper about the relationship between our model predictions and these reported cryoEM structures. We eventually come to the conclusion that this seeming discrepancy does not imply a fundamental contradiction, but rather arises from the distinction between the *functional* states defined in our model (namely, *o*, *h*, *c*) and the *structural* states captured by cryoEM (explained below). We did not make this

distinction clear enough in the original manuscript; in fact, we now realized our original use of term "predicted stable structures" may have been quite misleading.

We have now added a few sentences in the revised Results section to highlight this distinction (Page 4, highlighted in purple). We now also discuss how our model predictions may help interpret the functional roles of the cryoEM structures (Pages 18–20, highlighted in purple) and mention additional experimental evidence, while acknowledging nuances between our model variants.

Here is a summary of our line of arguments that we have added and what we have changed in the revision to properly explain this possible interpretation.
As now clarified in the revised Results section (Page 4, highlighted in purple), the conformational states *o*, *h*, *c* (c_1/c_2) of individual β-subunits are defined in our model as *functional* states: the *c* (or c_1/c_2) state is the catalytically active, high-affinity state; the *o* state is the functionally open, low-affinity state prior to ATP binding; the *h* state is the kinetic intermediate that connects the *o* and *c* states. These *functional* states can be tentatively identified with the *structural* states observed in the two cryoEM studies termed β_E/β_O, β_{HO}/β_{HC}, and $\beta_{TP}/\beta_{DP}/\beta_C$; however, such a direct, one-to-one association is not imposed in our Markov model, which is by design agnostic about any specific atomistic structures.

Consequently, what our model predicts (joint functional states of the three β-subunits in the 80°- and 120°-dwells, as listed in Table 4, Page 18) are not "stable structures", but rather a series of functional stages this molecular machine goes through in a catalytic cycle. We have added the new Supplementary Figure S9 to visualize these predicted functional stages (see panels a&b). At the beginning of the 80°-dwell (catalytic dwell), the enzyme first reaches an *hcc* state (the subscripts of c_1/c_2 are omitted), where the β_1-subunit awaits product dissociation and the β_2-subunit awaits ATP hydrolysis. After product dissociation in the β_1-subunit — but before ATP hydrolysis completes in the β_2-subunit, the enzyme transitions to an *occ* state. After ATP hydrolysis, the enzyme enters the 120°-dwell (ATP-waiting/binding dwell) and reaches an *ohc* state, where the β_1-subunit adopts the low-affinity *o* conformation and awaits substrate ATP binding. Subsequently and occasionally, if product dissociation occurs in the β_2-subunit before ATP binding in the β_1-subunit (more likely at very low ATP concentrations), the enzyme transitions to an *ooc* state.

From a thermodynamic perspective, our model predicts an ensemble of functional stages for each dwell. For the 80°-dwell, our model predicts the *hcc* stage to be most populated, while the *occ* stage exists as a transient state of lower population. For the 120°-dwell, our model predicts an ensemble of mainly *ohc* and *ooc* stages. In our original manuscript (Table 4), we claimed the *ooc* stage to be most populated. However, after further inspection, we found that the *ohc* stage can also be higher populated than *ooc* for some model parameters, without affecting the agreement with other measurements. Therefore, for higher similarity with the cryoEM structures (explained below), we now focus on the case where *ohc* is higher populated than *ooc*. In our revised manuscript (Table 4 and Supplementary Figure S9a&b), we now describe these predicted ensembles of functional stages more clearly.

How do these predicted functional stages compare with the reported cryoEM structures (see Supplementary Figure S9c–f)? Intuitively, one would expect a stage predicted of high population to be likely observed in experiments, so we first compare our model-predicted most populated stages (80°-*hcc*, 120°-*ohc*) with the cryoEM structures.

Notably, regarding the β_2- and β_3-subunits, our model predictions align with the Nakano and Sobti structures (by mapping β_{HO}-*h*, $\beta_{TP}/\beta_{DP}/\beta_C$-*c*), which also look very similar to each other.

However, regarding the β_1-subunit, the Nakano and Sobti structures seem to differ from each other, as they suggest two opposite scenarios for its conformational change as the γ-subunit rotates from 80° to 120°. In the Nakano structures, the catalytic interface of the β_1-subunit appears more compact at 80° than at 120° (although both are labeled β_E), suggesting a "further opening" scenario. On the contrary, in the Sobti structures, the β_1-subunit structure is less compact at 80° than at 120° (β_{HC} compared to β_O), suggesting a "partial closing" scenario. Notably, our model predictions (the β_1-subunit transitions from $h \rightarrow o$ upon the 80°→120° rotation) is consistent with the Nakano "further opening" scenario.

Which of these two scenarios is more plausible? In our added discussion (Pages 18–20, highlighted in purple), we now refer to additional experimental evidence in support of the "further opening" scenario, including recent nucleotide titration experiments (Y. Li *et al.*, *Arch. Biochem. Biophys.*, 2021, doi: 10.1016/j.abb.2021.108899; Ref. 22 in our revised manuscript). These experiments measured the nucleotide binding affinities of F_1-ATPase when the γ-subunit was stalled in the two dwells, and obtained lower affinities of the enzyme at 120° than at 80°. Specifically, these titration data demonstrated a remarkably low-affinity site (K_d > 5 mM) at 120°, corresponding to our *o* state.

In this "further opening" scenario, the Sobti structures may not necessarily correspond to the most populated stages predicted by our model, but instead to other, less populated stages, which would resolve the apparent contradiction. In fact, their 80°-dwell structure resembles the transient *occ* stage predicted by our model, and thus might have captured this post-product-dissociation stage. We consider it plausible that the use of a hydrolysis-slowing mutant in the cryoEM sample may have altered the kinetics such as to stabilized this stage sufficiently to be actually observed. Similarly, their 120°-dwell structure might represent a post-ATP-binding stage *h'hc* shortly after ATP binding, as the β_1-subunit begins to close (this *h'hc* stage is included by our model in the 200°-dwell ensemble, as a result of discretization of the γ-subunit rotation). This tentative scenario, which we have now added to the Discussion Section, would resolve the apparent discrepancy between the Sobti structures and the titration experiments, and would also provide a consistent mechanistic interpretation.

We note that despite these different interpretations of the β_1-subunit state, both our model predictions and these cryoEM structures are fully compatible with the cited single-molecule FRET study [Sugawa *et al.*, *PNAS* (2016); Ref. 61 in our revised manuscript], which is one of the very few experiments that allows simultaneous monitoring of γ-subunit rotation and β-subunit conformations. This study reports "2 closed + 1 non-closed" β-subunits at 80° and "1

closed + 2 non-closed” β-subunits at 120°. Whether the non-closed β-subunits are in an open or half-closed/half-open conformation, cannot be derived from this experiment. As of now, therefore, we are not aware of experimental data that are incompatible with our model predictions. Future studies will be required to finally resolve this issue.

We also thank the Reviewer for suggesting to modify our model such as to resolve the above apparent discrepancy. As we understand, the Reviewer suggests to restrict the *h* state in our model to product release, thus assuming different conformational pathways for ATP binding and product release, *e.g.*, *c*→*h*→*o* during product release, but *o*→*c* directly during ATP binding, similar to the scheme proposed by Sugawa *et al.* in their Fig. 7. Indeed, and remarkably, whereas our current model assumes the *h* state to be accessible in both processes (as the Reviewer correctly noted in Fig. 1e), these different conformational pathways are spontaneously and effectively reproduced by the fitted model parameters (free energies and free energy barriers): the *h* state is predicted to be a highly-populated intermediate during product release, but only a lowly-populated intermediate during ATP binding, thereby implementing Sugawa *et al.*’s scheme, without the need to enforce it. Therefore, we think it not necessary to restrict the *h* state in our model to product release process as an *a priori* assumption.

Finally, this comment, in line with Reviewer #1’s Comment (ii), highlights the importance of Pi release, and we refer to our reply to Reviewer #1 above. Briefly, we note that our model does not ignore this event; instead, and in the spirit of creating a minimal model, it describes the individual Pi and ADP release steps by a single, effective “product release” step, which is governed by the kinetics of the slower one of the two release events. We agree that future experiments may force us to explicitly distinguish between individual Pi and ADP release steps in more detailed F_1-ATPase models; at present, this does not seem to be the case.

4) In Conclusion, it was described “for a nucleotide-bound beta-subunit, the catalytically active, closed conformation is energetically more favorable than the open and partially conformations.” Where can I find the optimized values for supporting this?

Response: In reply to this question, we now provide the full distributions of the optimized values in Supplementary Figures S11–S14. However, to directly verify our conclusion from the distributions alone is not straightforward. Therefore, we refer the Reviewer also to a direct illustration of the free energies, presented in Figure 2f (Page 42) of the manuscript, and further detailed for all tested *ohc*-variants in Supplementary Section S2 (see particularly Supplementary Figure S7). To make this evidence more visible to readers, we have now added a direct reference to these figures to the corresponding sentence in the revised Discussion section (Page 17, highlighted in yellow).

Figure 2f compares the predicted free energies of a nucleotide-bound β-subunit in different conformations. For a nucleotide-bound β-subunit in a given conformation $\mathcal{C}$, its free energy consists of its conformational free energy $\Delta G_{\beta,\mathcal{C}}$ and its binding free energy $\Delta G^{\Theta}_{\mathrm{b},\mathcal{C},\mathcal{B}}$, where $\mathcal{B}$ =T, D for ATP/ADP-bound, respectively. Here, the model parameters $\Delta G_{\beta,\mathcal{C}}$ and $\Delta G^{\Theta}_{\mathrm{b},\mathcal{C},\mathcal{B}}$ are obtained from training against turnover rates, 100% chemo-mechanical coupling efficiency,

and nucleotide titration curves. As can be seen, all points lie above the diagonal line, indicating that for all trained parameter sets, the closed conformation is energetically more favorable.

To specifically test whether the 100% efficiency constraint is the key determinant for this property, we conducted an additional training for several *ohc*-variants using only the turnover rates and 100% efficiency as training data. The results, presented in Supplementary Figure S7 and discussed in detail in Supplementary Section S2, confirm that all parameter sets capable of reproducing the tight chemo-mechanical coupling share this property. This strongly supports our conclusion that the stability of the closed conformation is a necessary condition for the model to achieve high efficiency.

5) In equation (10), the minus sign on the right-hand side seems unnecessary.

Response: Many thanks for catching this typo. We have corrected it in the revised manuscript (now labelled Equation 13, Page 24, highlighted in yellow).

Point-by-point Responses (Second Revision)

(for manuscript "A Minimal Chemo-mechanical Markov Model for Rotary Catalysis of F_1-ATPase")

Below, the Reviewers' comments are quoted (text in blue), followed by our replies (text in black). In the revised manuscript, we have highlighted all corresponding changes.

Reviewer #1: The authors have addressed all my minor comments.
Reviewer #3: The authors have addressed all comments. Ready for publication.
Reviewer #4: The authors sufficiently addressed the points raised. I believe it is now publishable.

Response: We thank the Reviewers #1, #3, and #4 for approving our revised manuscript and recommending publication.

Reviewer #2

Thank you for your extensive explanations and the revision. But I am sorry that I was rather disappointed by your responses. As a natural scientist, I would pursue to understand what is going on in our world, specifically the principal principle working in such marvelous enzyme machines of living organisms. I appreciate your efforts to understand the mechanism of F-ATPase, but as I have commented, I am afraid that the research level of F-ATPase has not arrived to discuss its mechanism.

As you explain, the precise mechanisms of individual F-, V-, and A-ATPases would be different. But the principle of such energy transduction machineries would be expected to be the same. Scientists try to elucidate such general principles working in our world. If you are interested in the individual differences of them, you do not have to submit to Nat. Comm., a general science journal and rather should choose more specific journals.

What is known in V-ATPase mechanism (Arai et al., BBRC 533, 1413-1418 (2020)):

1) A and B subunits bind in the presence of nucleotides, especially with ATP; the affinity is weaker with ADP.
2) A or B alone has no ATP hydrolysis activity; A has ATP binding affinity.
3) A1B1 has high affinity for DF-axis; ATP (AMPPNP) stabilizes the A1B1DF complex strongly.
4) DF stabilizes A1B1 complex even without ATP.

Then we can speculate that A with ATP binds B, which in turn binds DF. A1B1DF hydrolyzes ATP to ADP, releasing Pi first in case of V-ATPase; A with ADP weakens the binding of B, thus of DF, releasing DF. DF floats thermally to the next ATP bound A1B1.

Is such mechanism known for F-ATPases? If so, then your research is valuable to understand the precise individual primary processes in the mechanism. Otherwise, it seems that your trials cannot specify the principal mechanism of the rotary ATPases. Therefore, I suggested that by including V-ATPase knowledges as universal principles of such rotary ATPases in the introduction section, the logics would become simpler and more easily understandable; just by renaming A, B, and DF to beta-, alpha-, and gamma-.

I suggest to address such principal principle of the rotary motors proposed for a specific V-ATPase as a precedent model, whether correct or wrong, and to try to discuss around such mechanism. Since I understood that the results in your section 4 and 5 seemed consistent with the proposed model, I expected that your research should provide great progress in our scientific knowledge. But your revision did not go into such discussion and just discussed about the specific individual processes, which shall give rather confusions in the scientific knowledge of rotary motors instead of giving development of clearer understanding what is going on in the rotary motors.

In this sense, I strongly oppose against publication of this manuscript as it is. I hoped that your works provide better understanding, but I am afraid that your manuscript as it is brings about only confusions in the research field of rotary motors.

Response: We thank the Reviewer for his/her conceptually motivated assessment of our work, which clearly helped us better understand his/her view than we did after the first round of reviews. We fully agree with the Reviewer that to pursue the principal principles of energy transduction across the rotary ATPase family is of fundamental importance. Following the advice of the Reviewer and the Editor, in our second revision we have substantially expanded the Introduction and Discussion sections to explicitly position our study within the broader context of rotary ATPases and the universal energy transduction principle (Pages 2–4, 18–19, 22, highlighted in yellow). In essence, we have formulated an additional line of arguments and discussion points that aims at reconciling the deterministic picture of chemo-mechanical coupling emphasized in the classical binding change paradigm with the inherent microscopic stochasticity of the rotary molecular motors that arises from fundamental physical principles. Specifically, we now emphasize an intrinsic thermal ratchet mechanism as the fundamental energy transduction principle, as suggested by the Reviewer and proposed in the work by Arai *et al.* [Arai *et al.*, *BBRC* 533, 1413-1418 (2020)].

Below, we provide a detailed explanation for our revisions.

Having carefully studied the work by Arai *et al.* (2020) pointed out by the Reviewer, we find our modeling approach to be very much in line with the conceptual framework proposed therein. Specifically, our Markovian approach rests on the notion that rotary catalysis is fundamentally

a stochastic process (a "thermal/Brownian ratchet") rather than a deterministic one (customarily termed a "power-stroke"). Importantly, ATP hydrolysis does not directly and deterministically generate a torque that drives the rotation of the central stalk, but rather induces "affinity changes" among subunits and thus modifies the free energy landscape in such a way that the inherent thermal fluctuations of the central stalk (the gamma-subunit in F-ATPase, or the DF axis in V-ATPase) are directionally rectified, allowing it to "float thermally" to the next binding site.

Indeed, this elegant, thermal-ratchet-like physical picture is exactly the fundamental mechanism that motivated our modeling efforts and that we aimed to test quantitatively in our study. As we now highlight in the revised Discussion (Page 18), our Markov model directly incorporates this picture by conceptualizing the rotations of the central stalk "as random thermal fluctuations which are neither directly nor deterministically coupled to ATP hydrolysis". Mechanistically, this is achieved in our model's network topology by executing mechanical rotations and ATP hydrolysis as separate, independent, and stochastic transition steps. Conversely, a power-stroke mechanism would be described by a Markov model different from ours, in which ATP hydrolysis and rotation would have been fused into a single concerted transition step.

Accordingly, we see our model as both a working example and a quantitative description of how the general energy transduction principle of rotary catalysis is executed at the molecular level in the specific case of F-ATPase. The fact that our model reproduces the available experimental data – ranging from macroscopic, deterministic data like the measured chemo-mechanical coupling efficiencies and titration curves to the microscopic, stochastic behaviors of the central stalk observed in the single-molecule experiments – demonstrates that a thermal ratchet mechanism as proposed by Arai *et al.* is both feasible and also sufficient to fully explain rotary energy transduction.

We thank the Reviewer for making us aware of this viewpoint, and now explicitly explain and discuss this important point in the revised manuscript. We now also mention that, by providing a specific and quantitative working example, the general energy transduction principle is markedly strengthened (Page 22). Particularly, we now also highlight in our discussion how the complex and highly stochastic rotations observed in single-molecule experiments at low ATP concentrations, also reproduced by our model in the kinetic Monte-Carlo simulations, strongly favor the Brownian ratchet mechanism. Indeed, a thermal ratchet mechanism seems to be the most straightforward and intuitive explanation for the observed back-and-forth, irregular rotations which can accumulate up to several revolutions in one direction. Should F-ATPase behave like an intrinsic power-stroke, we would expect, based on the mechanics of the alternatively designed Markov model, that the rotational trajectories would look very different at low ATP concentrations from the observed ones, exhibiting long, static dwells followed by rare, strict unidirectional steps triggered by occasional ATP hydrolysis.

To facilitate this new line of arguments, and also to follow the Reviewer's specific suggestions, we now cite the work by Arai *et al.* and a closely related review paper (Refs. 29&30 in our revised manuscript), linking our modeling approach of an intrinsic thermal-ratchet mechanism

to the “affinity change” framework developed therein (Page 18–19). As suggested by the Reviewer, we now also explicitly discuss the V-ATPase mechanism in the Introduction and highlight the structural and functional homology between the subunits, mapping the alpha-, beta- and gamma-subunits in F-ATPase to their V/A-ATPase counterparts (B, A, and DF) (Page 2).

We believe this revision not only better explains how our quantitative approach supports the universal principles of rotary energy transduction as the Reviewer suggested, but also widens the conceptual scope of our paper by presenting a quantitative and thermodynamically consistent implementation of these principles.

Point-by-point Responses (Third Revision)

(for manuscript "A Minimal Chemo-mechanical Markov Model for Rotary Catalysis of F_1-ATPase")

We thank Reviewer #2 for his/her kind words and now very positive assessment and approval of our revision. We would also like to thank for his/her thoughtful comments, which have enabled us to considerably strengthen the scope of our manuscript, as well as the Editor for her helpful support.

Below, the Reviewer's comments are quoted (text in blue), followed by our replies (text in black). In the revised manuscript, we have highlighted all corresponding changes.

We hope our manuscript now meets the standard of Nature Communications and look forward to your reply.

Best regards

Helmut Grubmüller

Reviewer #2

Thank you very much for your inclusive revision. I am happy to know that you understood my proposal of research attitude toward science. I fully agree with your comment aiming to elucidate the working principles under biological rotary motors.

Touching about the precedent V-ATPase affinity change mechanism in introduction section made the logic clearer, simpler, and easily understandable. I appreciate your revisions in introduction and discussion.

At first stage of reviewing, I expected to shorten or to eliminate the 3-conformation model, because this part was not so much relevant to the principle of the mechanism and made the manuscript long and complicated. You kept this part in your previous revision, but I opposed against publication from different but more basic reason. Now since you agreed to include the V-ATPase affinity change mechanism in the introduction section, would you please reconsider this point? Other reviewers have agreed with publication as it was, and this point is not essential nor fatal for the improvement of the manuscript, therefore, I do not oppose against publication as it is, but I suggest you to reconsider whether extensive explanation of 3-variants model may bring about some confusion in readers.

Otherwise, I appreciate your revision and your present work. This must give researchers more profound and further progress in understanding the universal principle of the rotary motor mechanism.

Response: We thank the Reviewer for his/her positive and appreciative comments and thoughtful feedback. We also appreciate and understand the Reviewer's suggestion regarding the readability of the extensive discussion of the 3-conformation model in our manuscript. To address remaining concerns that this subsection might still cause some confusion on the readers' side, we have now further streamlined the text by removing some overly technical details and adding clear signposts to better guide the logical flow (Pages 7–10; strengthened signposts are highlighted in yellow; shortened paragraphs are highlighted in purple). The word count of the two relevant subsections is reduced by almost one third, from ~1800 to ~1200.

Having carefully discussed the pros and cons of presenting a discussion of the 3-conformation model, we have decided to retain the main structure of this analysis, and not to eliminate it, as suggested by the Reviewer. The reason is as follows. As highlighted in our Discussion, we consider it a core methodological contribution of our work to objectively assess and compare competing mechanistic hypotheses against available experimental data in a thermodynamically consistent manner. Here, the 3-conformation and 4-conformation models represent the primary competing hypotheses evaluated through this approach. Only after the detailed evaluation of the 3-conformation variants can one demonstrate definitively that a model with only three beta-subunit conformations is fundamentally incompatible with the known properties of F_1-ATPase. Thus, we feel that presenting this rigorous exclusion is a necessary logical step in our analysis, demonstrating to the readers that our transition to the 4-conformation model (ohc_1c_2-variants) is a data-driven necessity rather than an arbitrary assumption. We therefore also feel that omitting this step would compromise the transparency of our methodology.

We hope the Reviewer can agree that with the new textual refinements to prevent confusion and reader fatigue, we have struck the right balance between maintaining a rigorous and logically consistent scientific flow of arguments and good readability. We are very grateful for the Reviewer's constructive dialogue throughout the review process.
